# Phaeochromycins F–H, three new polyketide metabolites from *Streptomyces* sp. DSS-18

**DOI:** 10.3762/bjoc.4.46

**Published:** 2008-12-02

**Authors:** Jian Li, Chun-Hua Lu, Bao-Bing Zhao, Zhong-Hui Zheng, Yue-Mao Shen

**Affiliations:** 1 Key Laboratory of the Ministry of Education for Cell Biology and Tumor Cell Engineering; Xiamen Engineering Research Center of Marine Microbial Drug Discovery; Fujian Engineering Laboratory for Pharmaceuticals; School of Life Sciences, Xiamen University, Xiamen, Fujian 361005, P. R. China; 2 School of Life Sciences, Xiamen University, No. 422 South Siming Road, Xiamen, Fujian 361005, China (address for correspondence); Phone: 86-592-2181722, Fax: 86-592-2181722

**Keywords:** biological properties, isolation, polyketides, Streptomyces, structure determination

## Abstract

Three new polyketides, phaeochromycins F (**1**), G (**2**), H (**3**), were obtained from the culture broth of marine actinomycete strain *Streptomyces* sp. DSS-18. Their structures were established on the basis of detailed spectroscopic analyses, including 1D-, 2D-NMR and HR-ESI MS techniques.

## Introduction

Marine microorganisms are widely recognized as rich sources of novel natural products [1–2]. In recent years, numerous novel compounds discovered from marine actinomycetes have been reported [3–5]. During the course of our search for biologically active substances from marine derived actinomycetes, a strain of the genus *Streptomyces* was isolated from deep sediment collected from the west Pacific.

**Figure 1:**
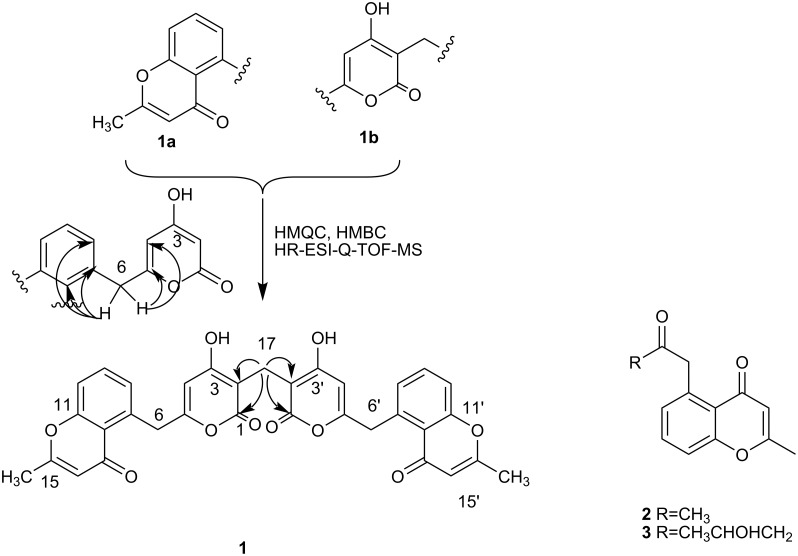
The fragments **1a** and **1b** of compounds **1** and compound **2**, **3** and the selected HMBC correlations (H→C).

Herein, we report the isolation and structure determination and biological properties of three new polyketides, namely phaeochromycins F (**1**), G (**2**) and H (**3**), from *Streptomyces* sp. DSS-18 (see Figure 1).

## Results and Discussion

Fermentation was carried out at 28 °C for 2 weeks with aeration (10 L/min) under constant agitation (240 rpm). After filtration of the harvested culture broth, the culture filtrate was extracted exhaustively with ethyl acetate. The ethyl acetate extract was purified by column chromatography (RP-18, Sephadex LH-20, and silica gel) to afford three new polyketides.

Phaeochromycin F (**1**) was obtained as a brown powder. The IR absorption at 3424 and 1653 cm^−1^ indicated the presence of OH groups and aryl ketone. The ^1^H NMR spectra of **1** (Table 1) showed a Me signal [δ(H) 2.34 (s)], two CH_2_ moieties [δ(H) 4.53 (s), 3.43 (s)], five aromatic CH groups [δ(H) 5.80, 6.06, 7.18, 7.57, 7.39]. The ^13^C NMR (DEPT) spectrum of **1** (Table 1) showed 17 signals: one Me, two CH_2_, five CH, and nine quaternary carbons. The aromatic protons at δ 7.18 (d, *J* = 7.5 Hz), 7.57 (dd, *J* = 7.5, 8.5 Hz), 7.39 (d, *J* = 8.5 Hz) suggested the presence of a trisubstituted aromatic ring. The HMBC correlations from H-C(8) to C(5), C(6), C(7), C(9), C(10), C(11), C(12) and C(13), and H-C(9) to C(7), C(8), C(10), C(11) and C(12), and from H-C(10) to C(7), C(8), C(9), C(11), C(12) and C(13), and from H-C(14) to C(12), C(13), C(15) and C(16), along with ^1^H,^1^H-COSY correlations, and the connection from C(11) to C(15) via oxygen was confirmed by downshift of their chemical shift, established the presence of fragment **1a** (benzopyranone). The assignments of the benzopyranone carbons are in good agreement with those of SEK34b [6]. Fragment **1b** was deduced based on the long-range correlations from H-C(4) to C(2), C(3), C(5), C(6) and the hydroxy proton at δ 10.67 to C(2), C(3) and C(4), and their chemical shift (Table 1). Based on the long range correlations from H-C(6) to C(4), C(5), C(7), C(8) and C(12), fragments **1a** and **1b** were joined together (Figure 1). The HMBC correlations from H-C(17) to C(1) and C(2) suggested a symmetrical structure, with symmetry center at C(17). The integral ratio of two methylene groups (CH_2_-6 to CH_2_-17) is 2:1, this also indicated that the symmetry center was at C(17). Its HR-ESI mass spectrum showed the [M + Na]^+^ peak at *m/z* 603.1282, establishing the molecular formula C_33_H_24_O_10_, which further supported the symmetrical structure. Therefore, from those data, the structure of **1** was elucidated as 5-{[4-hydroxy-3-({4-hydroxy-6-[(2-methyl-4-oxo-4*H*-chromen-5-yl)methyl]-2-oxo-2*H*-pyran-3-yl}methyl)-2-oxo-2*H*-pyran-6-yl]methyl}-2-methyl-4*H*-chromen-4-one, named phaeochromycin F.

**Table 1:** ^1^H, ^13^C NMR data of **1** at 500, 125 MHz, in CDCl_3_, δ in ppm, *J* in Hz.

Position	^1^H	^13^C

1/1′	–	169.8 (s)
2/2′	–	101.7 (s)
3/3′	–	168.6 (s)
4/4′	5.80 (s)	102.4 (d)
5/5′	–	163.6 (s)
6/6′	4.53 (s)	37.8 (t)
7/7′	–	135.9 (s)
8/8′	7.18 (d, *J* = 7.5)	128.5 (d)
9/9′	7.57 (dd, *J* = 7.5, 8.5)	132.9 (d)
10/10′	7.39 (d, *J* = 8.5)	118.1 (d)
11/11′	–	158.0 (s)
12/12′	–	121.4 (s)
13/13′	–	179.3 (s)
14/14′	6.06 (s)	111.8 (d)
15/15′	–	164.8 (s)
16/16′	2.34 (s)	20.1 (q)
17	3.43 (s)	18.5 (t)
OH	10.67 (s)	

Phaeochromycin G (**2**) was isolated as an amorphous powder. Its molecular formula was determined as C_13_H_12_O_3_ based on HR-ESI mass spectra and NMR data. The IR absorption at 1660 cm^−1^ indicated the presence of aryl ketone. Inspection of the NMR spectral data (proton, carbon, DEPT, HMBC) (Table 2) indicated that **2** had the same fragment **1a**. The long-range correlations from H-C(2) to C(3) and C(4) and H-C(4) to C(3), C(5), C(6) and C(10) suggested the presence of the acetonyl group connected to C(5). Again, NMR assignments were in excellent agreement with the literature [7]. Thus, the structure of **2** was determined as 2-methyl-5-(2-oxopropyl)-4*H*-chromen-4-one.

**Table 2:** ^1^H, ^13^C NMR Data of **2** and **3** at 600, 150 MHz, in CDCl_3_, δ in ppm, *J* in Hz.

	**2**		**3**	

Position	^1^H	^13^C	^1^H	^13^C

1				
2	2.38 (s)	30.1 (q)	1.28 (d, *J* = 6.0)	22.5 (q)
3	–	205.1 (s)	4.39 (m)	64.3 (d)
4	4.27 (s)	49.6 (t)	2.81 (dd, *J* = 9.6, 16.8)	51.1 (t)
5	–	136.4 (s)	2.90 (dd, *J* = 2.4, 16.8)	206.6 (s)
6	7.05 (d, J = 7.2)	128.7 (d)	–	49.8 (t)
7	7.56 (t, J = 7.8)	132.7 (d)	4.22 (d, *J* = 16.8)	135.7 (s)
8	7.37 (d, J = 8.4)	117.5 (d)	4.36 (d, *J* = 16.8)	128.8 (d)
9	–	158.0 (s)	–	132.8 (d)
10	–	121.2 (s)	7.07 (d, *J* = 7.2)	117.7 (d)
11	–	179.6 (s)	7.60 (dd, *J* = 7.2, 9.0)	157.9 (s)
12	6.07 (s)	111.5 (d)	7.41 (d, *J* = 9.0)	121.4 (s)
13	–	165.3 (s)	–	179.5 (s)
14	2.33 (s)	20.2 (q)	–	111.5 (d)
15			–	165.2 (s)
16			6.09 (s)	20.1 (q)

Phaeochromycin H (**3**) was isolated as white powder. Its molecular formula was determined as C_15_H_16_O_4_ based on HR-ESI mass spectra and NMR data. Inspection of the NMR spectral data (proton, carbon, DEPT, HMBC) (Table 2) indicated that **3** had the same fragment **1a**. The ^1^H,^1^H-COSY clearly demonstrated the connectivity from H-C(2) to H-C(4); further analysis of HMBC also supported the connections. The HMBC correlations of H-C(4) to C(2), C(3), C(5), C(6) and H-C(6) to C(5), C(7), C(8) and C(12) suggested connectivity from C(4) to C(6) and C(6) to C(7). The remaining hydroxyl moiety dictated by the molecular formula of **3** was easily accommodated at C(3) (δ 64.3) and is supported by the observed chemical shifts of C(2) and C(3). From a comparison of the NMR data with those of phaeochromycin D [7], the structure of compound **3** was elucidated as 5-(4-hydroxy-2-oxopentyl)-2-methyl-4*H*-chromen-4-one.

Symmetrical and asymmetrical natural products linked by a saturated methylene group such as phaeochromycin F are rare.

### Bioassays

Graziani et al. [7] reported that phaeochromycins A–E are anti-inflammatory compounds which inhibit MAPKAP-2 kinase for use as leads in the development of new agents for treating rheumatoid arthritis. In our studies, cytotoxicities of phaeochromycins F–H were investigated using the HeLa cell line, following the MTT standards [8] and using cisplatin (DDP) as a positive control. Phaeochromycins F and G were found to have weak cytotoxicities with inhibitory rates 9.4% and 1.0% at a concentration of 10 μg/ml. Phaeochromycin H showed a modest inhibitory rate of 46.0% at a concentration of 10 μg/ml.

## Experimental

### General

Precoated TLC plates (silica gel G; Qingdao Marine Chemical Factory, Qingdao, P. R. China). For column chromatography (CC), silica gel (200–300, and 80–100 mesh; Qingdao), silica gel GF_254_ (Merck), RP-18 gel (Merck), and Sephadex LH-20 gel (Amersham Biosciences) were used. UV Spectra: UNICO single-beam 210A spectral photometer; 190–1100 nm, in MeOH. Optical rotations were obtained on a Perkin-Elmer 341 polarimeter with CHCl_3_ as solvent. The IR spectra were measured in KBr on a Nicolet FT-IR 360. NMR Spectra: Bruker DRX-500 and AV-600 instruments; δ in ppm rel. to Me_4_Si, *J* in Hz. MS: Bruker HR-ESI mass spectrometer; in *m/z*.

### Fermentation and Isolation of the Strain

The strain *Streptomyces* sp. DSS-18 was isolated from the sediment collected from West Pacific Ocean and was identified as *Streptomyces* sp. according to its 16S rDNA sequence (bankit1156038 FJ472840). Genomic DNA of strains was obtained by sodium dodecyl sulfate (SDS)-proteinase K lysis, selective precipitation of cell debris and polysaccharides with CTAB (hexadecyltrimethylammonium bromide), and isopropanol precipitation [9]. The bacterial strain was incubated on slope of GS media in a test tube at 28 °C for 5 d to afford seed cultures. After 48 h on a rotary shaker (180 rpm, 28 °C) 10 L of the preculture was fermented in a 150 L fermentor containing 100 L of sterilized GS medium. Fermentation was carried out at 28 °C for 2 weeks with aeration (10 L/min) under constant agitation (240 rpm).

### Extraction and Isolation

After filtration of the harvested culture broth of strain DSS-18, the culture filtrate was extracted with ethyl acetate. The ethyl acetate extract was partitioned between petroleum ether and methanol. The methanol solution was collected and evaporated to dryness *in vacuo* to afford 30 g of extract. The extract (30 g) was subjected to MPLC (130 g, RP-18), and eluted with H_2_O, and 30, 50, 70, and 100% acetone, respectively 2 L each, yielded 4 fractions: *Fr. S1-S4*. *Fr. S2* (4.3 g) was subjected to MPLC (130 g, RP-18), eluting with H_2_O, and 30, 50, 70, and 100% MeOH, respectively (2 L each) to yield 4 fractions; *Fr. S2a-S2d. Fr. S2c* (990 mg) was subjected to CC (100 g Sephadex LH-20; MeOH). All fractions were analyzed by TLC (CHCl_3_/MeOH 8:1), and pooled into five portions (*Fr. S2c1-S2c5*). *Fr. S2c4* (57 mg) was further purified by CC (silica gel, petroleum ether/ethyl acetate 5:1) to yield **1** (22 mg). *Fr. S1* (13.2 g) was subjected to MPLC (130 g, RP-18), eluting with H_2_O, and 20, 40, 70 and 100% MeOH, respectively. 2 L each yielded 5 fractions: *Fr. S1a-S1e. Fr. S1c* (6.4 g) was subjected to CC (100 g Sephadex LH-20; MeOH). All fractions were pooled into six portions (*Fr. S1c1-S1c6*). *Fr. S1c3* (660 mg) was further purified by CC (silica gel, CHCl_3_/MeOH 100:1) to **2** (51 mg), and *Fr. S1c3b* (30 mg) which was further purified by CC (silica gel, Petroleum ether/Ethyl acetate 3:1) and repeated MPLC (RP-18, 40% MeOH) to yield **3** (10 mg).

#### Phaeochromycin F

(5-{[4-hydroxy-3-({4-hydroxy-6-[(2-methyl-4-oxo-4*H*-chromen-5-yl)methyl]-2-oxo-2*H*-pyran-3-yl}methyl)-2-oxo-2*H*-pyran-6-yl]methyl}-2-methyl-4*H*-chromen-4-one **1**), brown, amorphous powder. UV (MeOH): 301.5; [α]*_D_*^20^ = 0 (*c* = 0.5, CHCl_3_); IR (KBr): 3424, 2974, 1653, 1391, 1089, 1050; ^1^H and ^13^C NMR: see Table 1; HR-ESIMS: *m/z* 603.1282 ([*M* + Na]^+^; calc. 603.1267).

#### Phaeochromycin G

[2-methyl-5-(2-oxopropyl)-4*H*-chromen-4-one; **2**], amorphous powder; UV (MeOH): 248, 300; [α]*_D_*^20^ = 0 (*c* = 0.6, CHCl_3_); IR (KBr): 1660, 1603, 1391, 1118; ^1^H and ^13^C NMR: see Table 2; HR-ESIMS: *m/z* 217.0897 ([*M* + H]^+^ calc. 217.0859).

#### Phaeochromycin H

[5-(4-hydroxy-2-oxopentyl)-2-methyl-4*H*-chromen-4-one; **3**], white powder; UV: 230, 249.5, 302; [α]*_D_*^20^ = +13.2 (*c* = 0.6, CHCl_3_); IR: 3438, 2971, 1712, 1644, 1605, 1392, 1366, 1118; ^1^H and ^13^C NMR: see Table 2; HR-ESIMS: *m/z* 283.2717 [*M* + Na]^+^ calc. 283.2749).

## Supporting Information

File 1 NMR spectra of compounds **1**–**3**

## References

[1] Bull A T, Ward A C, Goodfellow M (2000). Microbiol Mol Biol Rev.

[2] Fenical W (1993). Chem Rev.

[3] Charan R D, Schlingmann G, Janso J, Bernan V, Feng X, Carter G T (2004). J Nat Prod.

[4] Lee H-S, Shin H J, Jang K H, Kim T S, Oh K-B, Shin J (2005). J Nat Prod.

[5] Mitchell S S, Nicholson B, Teisan S, Lam K S, Potts B C M (2004). J Nat Prod.

[6] McDaniel R, Ebert-Khosla S, Hopwood D A, Khosla C (1994). J Am Chem Soc.

[7] Graziani E I, Ritacco F V, Bernan V S, Telliez J-B (2005). J Nat Prod.

[8] Mosmann T (1983). J Immunol Methods.

[9] Wilson K, Ausubel F M, Brent R, Kingston R E (1987). Preparation of genomic DNA from bacteria. Current protocols in molecular biology.

