# Supplementary material for: Phaeochromycins F–H, three new polyketide metabolites from Streptomyces sp. DSS-18
Source: Beilstein J Org Chem. 2008 Dec 2;4:46. doi: 10.3762/bjoc.4.46 (PMC2633662; doi:10.3762/bjoc.4.46)
Supplement: File 1 — NMR spectra of compounds 1–3 [file Beilstein_J_Org_Chem-04-46-s001.pdf]

Phaeochromycins F–H, three new polyketide metabolites from *Streptomyces* sp. DSS-18

Jian Li, Chun-Hua Lu, Bao-Bing Zhao, Zhong-Hui Zheng* and Yue-Mao Shen

Index

Page SI 2–18 NMR spectra of compounds **1–3**.

Page SI 19 HPLC data of compound **1** and crude extract.

1-1H

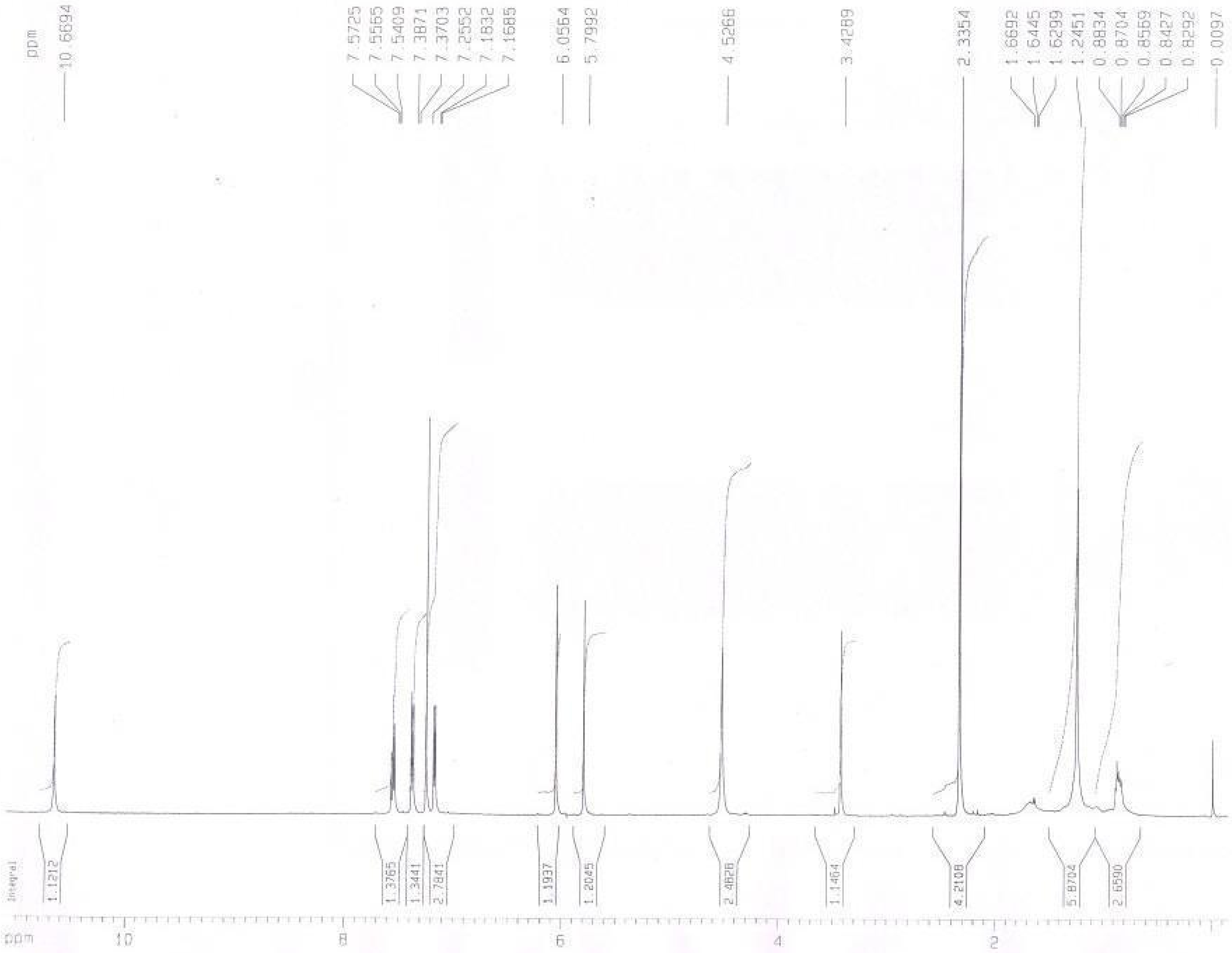

Current Data Parameters
NAME mds20
EXPNO 21
PROCNO 1

F2 - Acquisition Parameters
Date_ 20051125
Time 17.17
INSTRUM DRX500
PROBHD 5 mm BBI 1H-BB
PULPROG zg
TD 32768
SOLVENT CDCl3
NS 1
DS 0
SWH 6127.451 Hz
FIDRES 0.186995 Hz
AQ 2.6739187 sec
RG 80.6
DW 81.600 usec
DE 6.00 usec
TE 300.0 K
D1 2.00000000 sec

======== CHANNEL f1 ========
NUC1 1H
P1 10.00 usec
PL1 -4.00 dB
SFO1 500.1329931 MHz

F2 - Processing parameters
SI 16384
SF 500.1300160 MHz
WDW EM
SSB 0
LB 0.30 Hz
GB 0
PC 1.00

1D NMR plot parameters
CX 22.00 cm
CY 14.00 cm
F1P 11.095 ppm
F1 5548.92 Hz
F2P -0.152 ppm
F2 -75.93 Hz
PPMCM 0.51122 ppm/cm
HZCM 255.67503 Hz/cm

1-DEPT

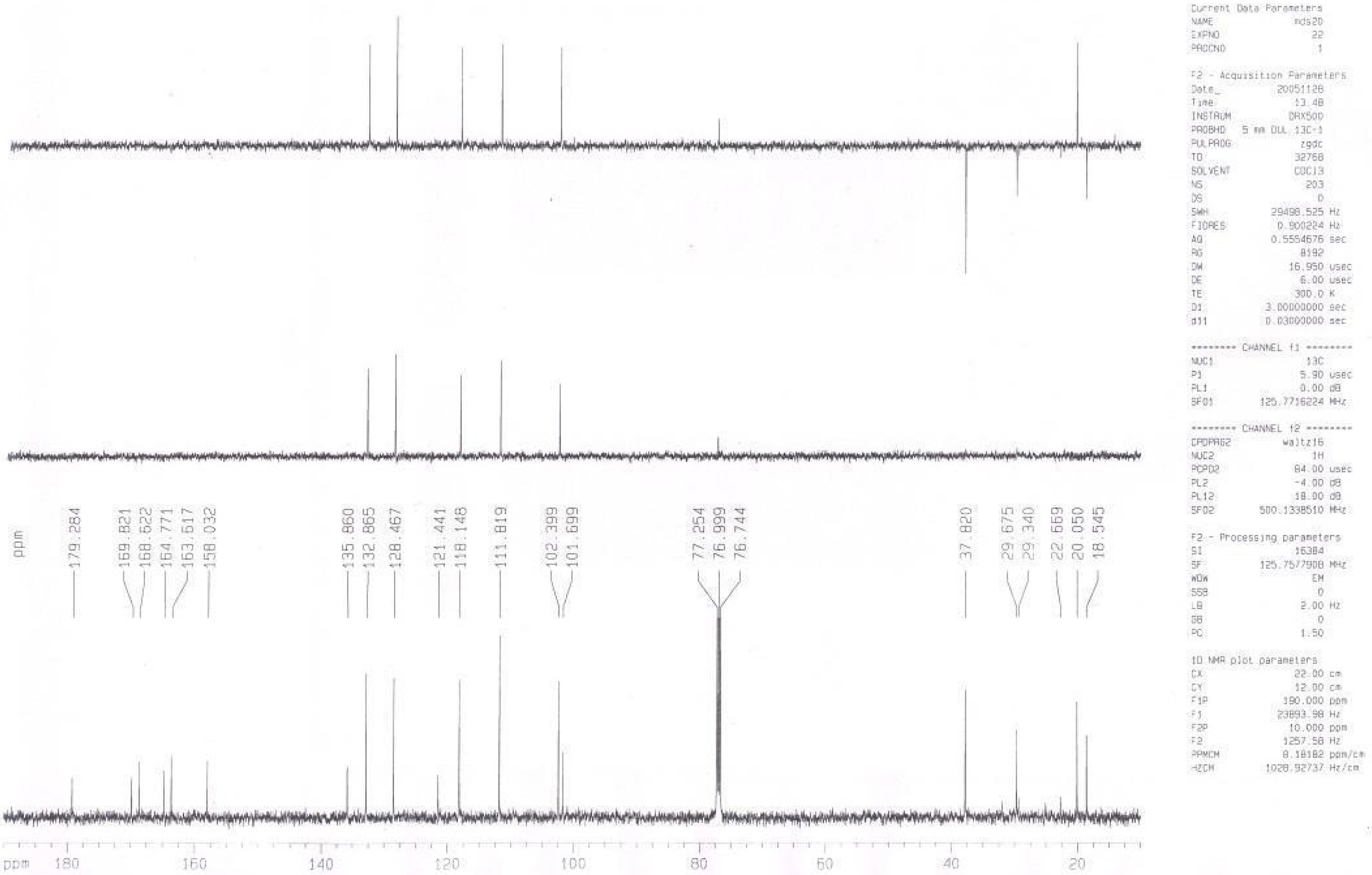

ppm
179.284
169.821
168.522
164.771
163.517
158.032
135.860
132.865
128.467
121.441
118.148
111.819
102.399
101.699
77.254
76.999
76.744
37.820
29.675
29.340
22.669
20.050
18.545
ppm 180 160 140 120 100 80 60 40 20
Current Data Parameters
NAME mds20
EXPNO 22
PROCNO 1
F2 - Acquisition Parameters
Date_ 20051128
Time 13.48
INSTRUM DRX500
PROBHD 5 mm DUL 13C-1
PULPROG zgdc
TD 32768
SOLVENT CDCl3
NS 203
DS 0
SWH 29498.525 Hz
FIDRES 0.900224 Hz
AQ 0.5554676 sec
RG 8192
DW 16.950 usec
DE 6.00 usec
TE 300.0 K
D1 3.00000000 sec
d11 0.03000000 sec
======== CHANNEL f1 ========
NUC1 13C
P1 5.90 usec
PL1 0.00 dB
SFO1 125.7716224 MHz
======== CHANNEL f2 ========
CPDPRG2 waltz16
NUC2 1H
PCPD2 84.00 usec
PL2 -4.00 dB
PL12 18.00 dB
SFO2 500.1338510 MHz
F2 - Processing parameters
SI 16384
SF 125.7577908 MHz
WDW EM
SSB 0
LB 2.00 Hz
GB 0
PC 1.50
1D NMR plot parameters
CX 22.00 cm
CY 12.00 cm
F1P 190.000 ppm
F1 23893.98 Hz
F2P 10.000 ppm
F2 1257.58 Hz
PPMCM 8.18182 ppm/cm
HZCM 1028.92737 Hz/cm

1-HSQC

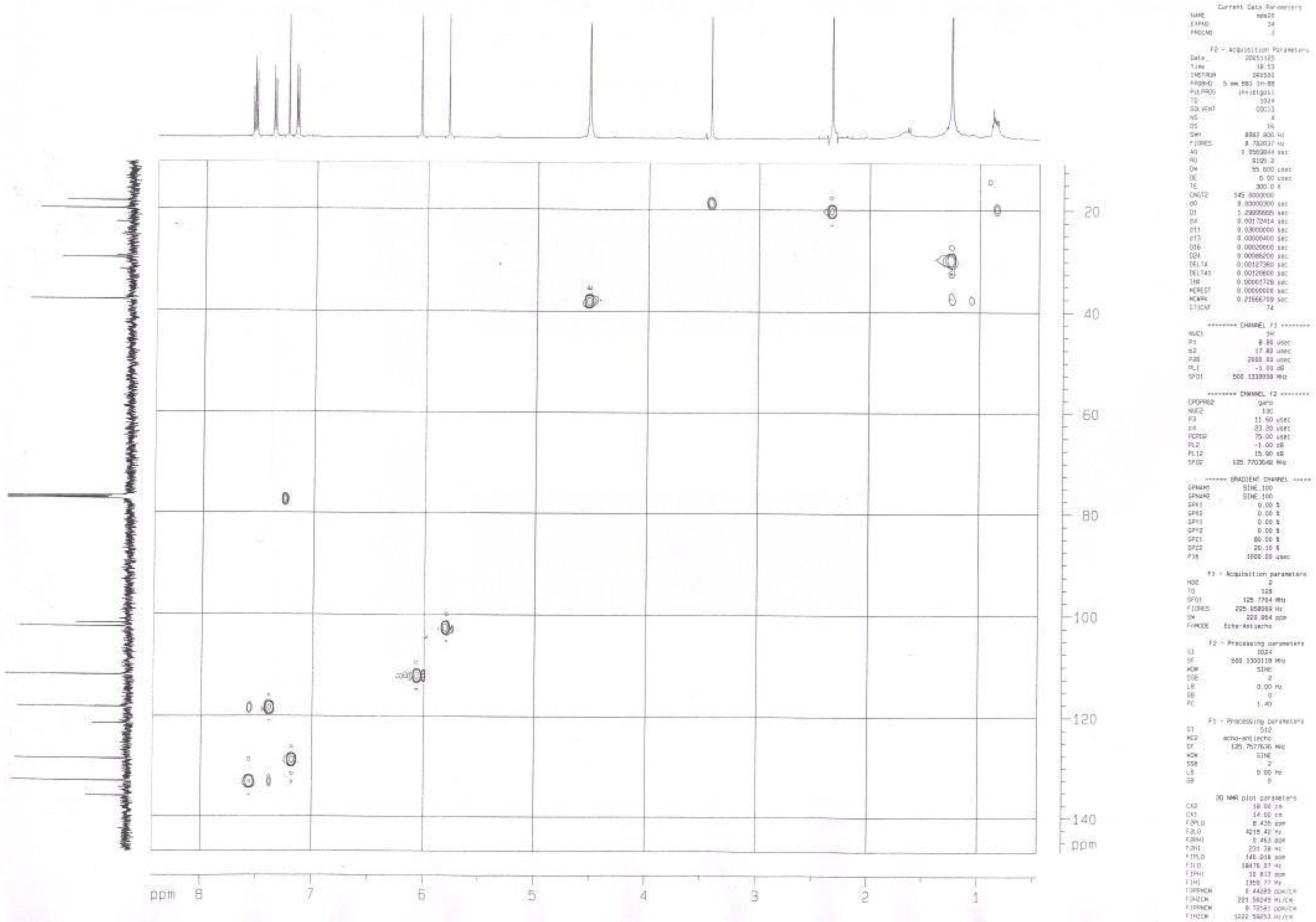

1-HMBC

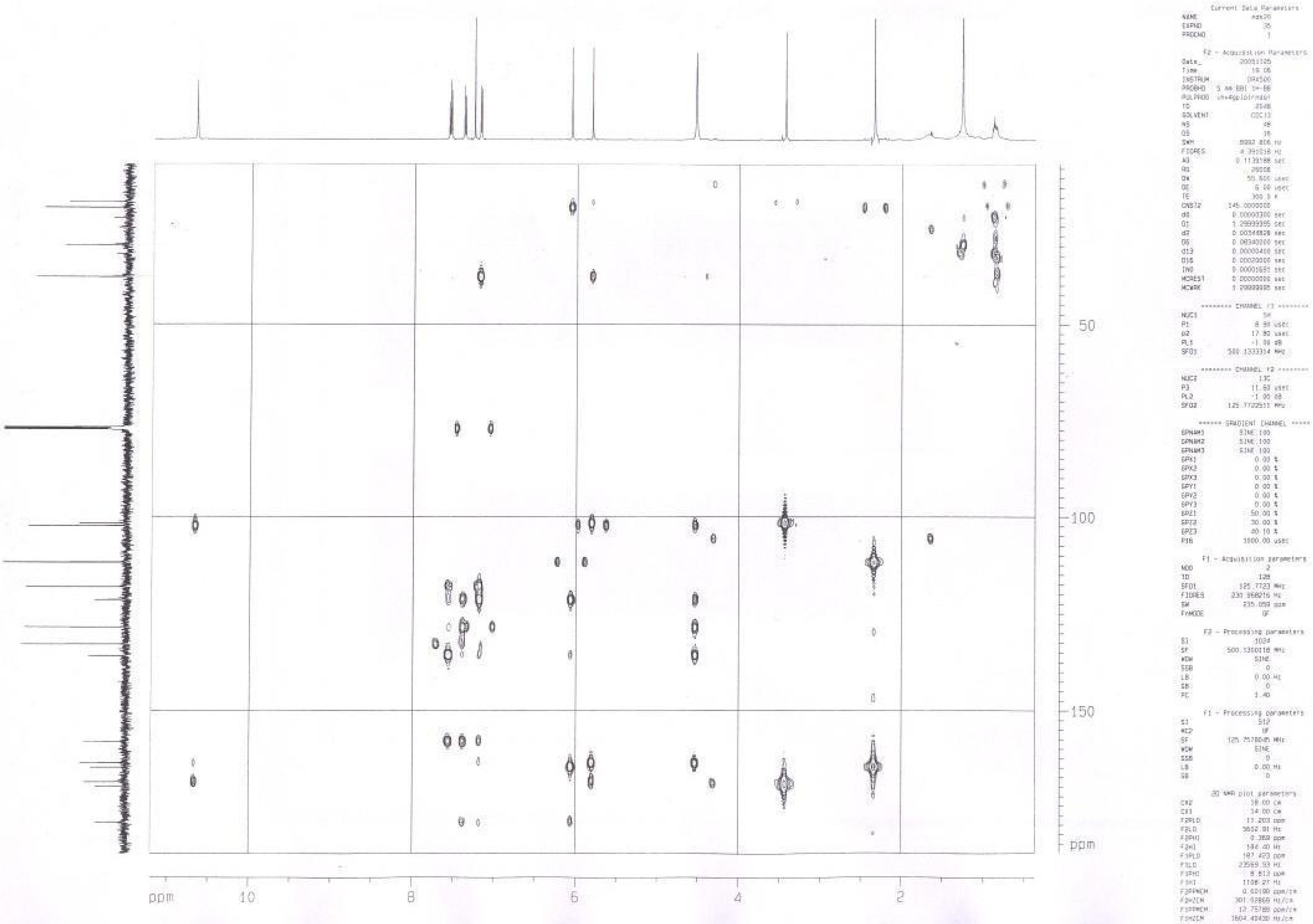

1-HMBC

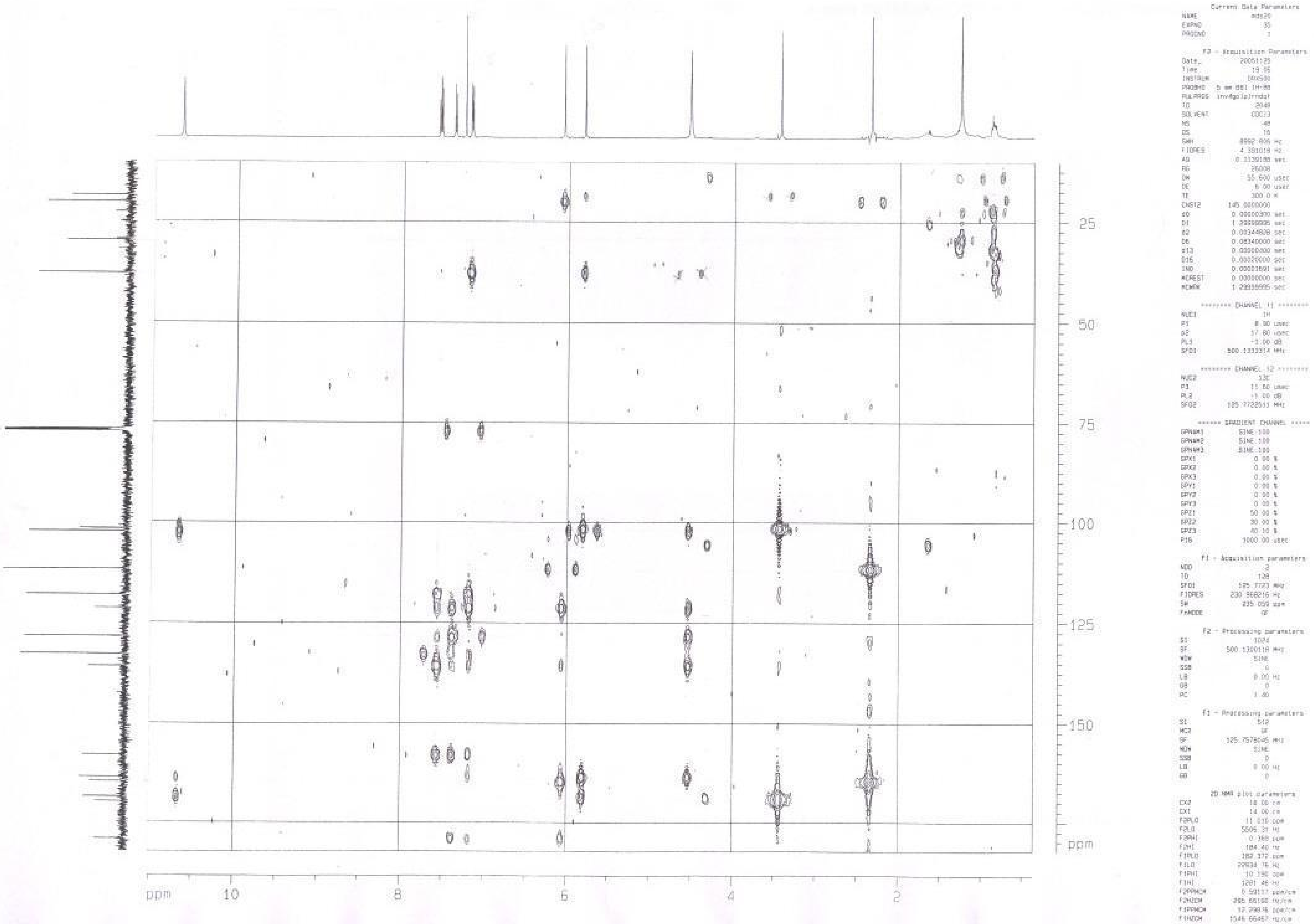

1-HMBC

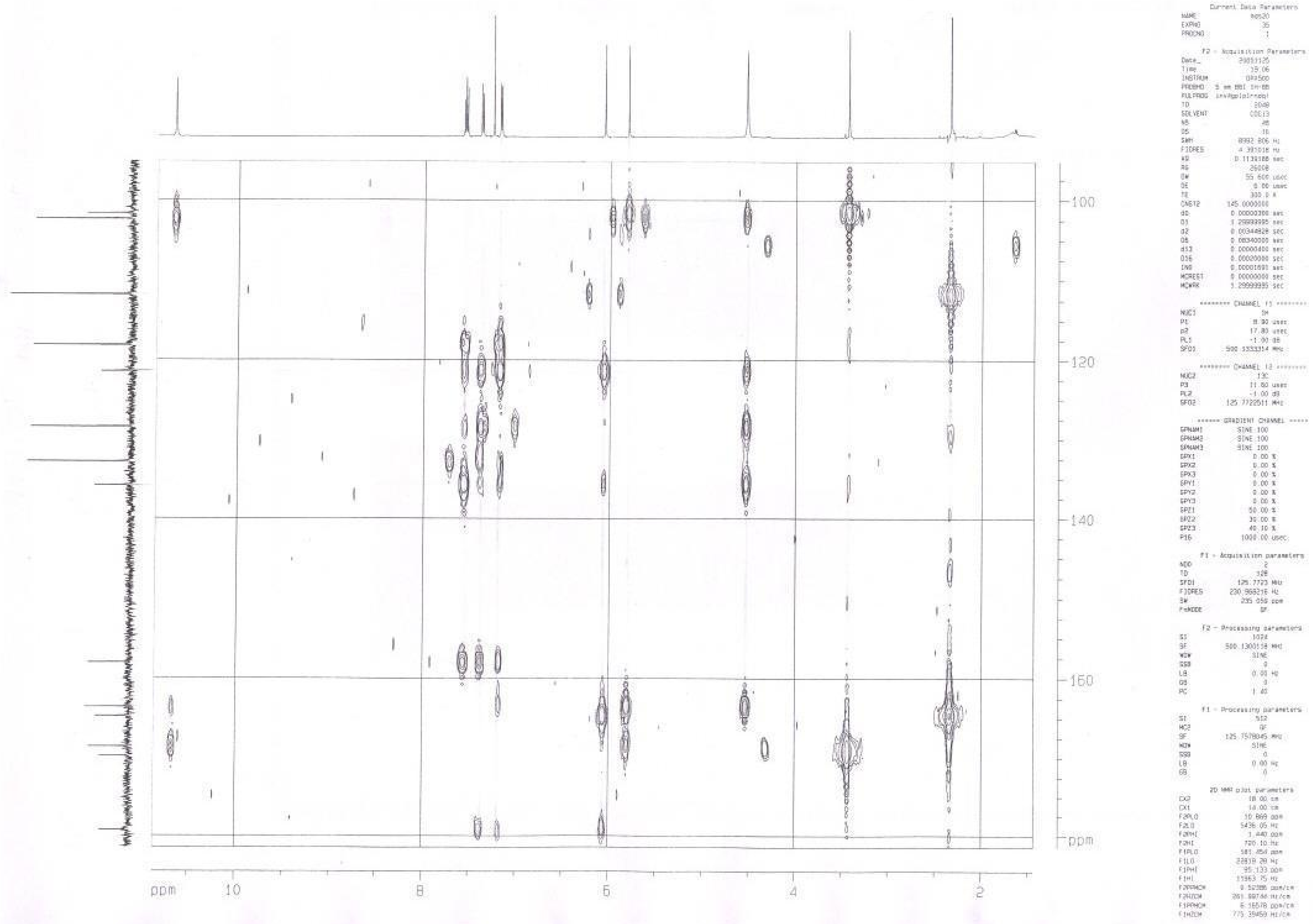

1- H-H- COSY

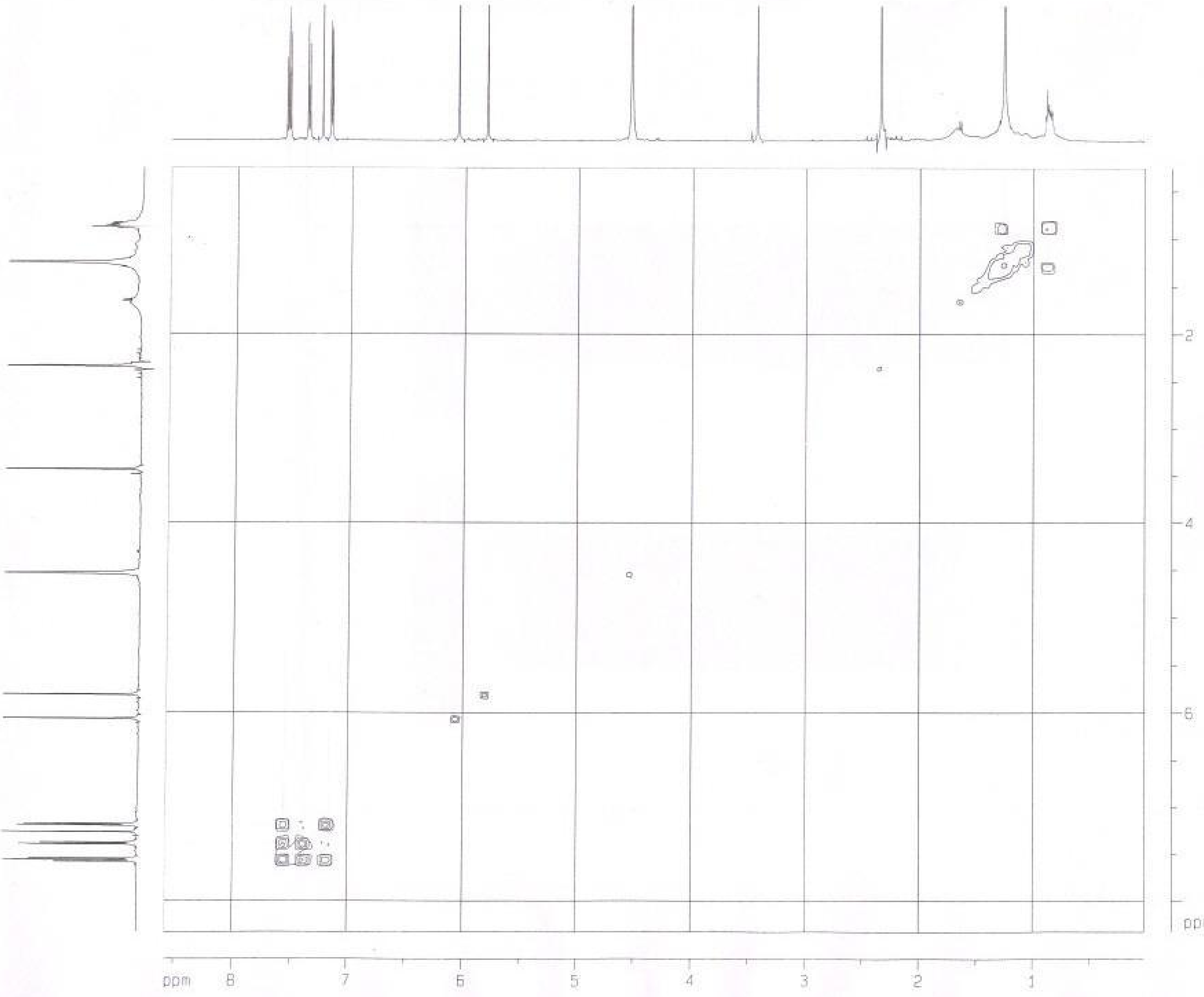

Current Data Parameters
NAME mds20
EXPNO 33
PROCNO 1

F2 - Acquisition Parameters
Date_ 20051125
Time 18.43
INSTRUM DRX500
PROBHD 5 mm BBI 1H-BB
PULPROG cosygpmfqf
TD 1024
SOLVENT CDCl3
NS 2
DS 16
SWH 8012.820 Hz
FIDRES 7.825020 Hz
AQ 0.0639476 sec
RG 36008
DW 62.400 usec
DE 6.00 usec
TE 300.0 K
D0 0.00000300 sec
D1 2.00000000 sec
d13 0.00000400 sec
D16 0.00020000 sec
IN0 0.00012496 sec
MCREST 0.00000000 sec
MCWRK 2.00000000 sec

======== CHANNEL f1 ========
NUC1 1H
P1 8.90 usec
PL1 -1.00 dB
SFO1 500.1318310 MHz

====== GRADIENT CHANNEL =====
GPNAM1 sine.100
GPNAM2 sine.100
GPNAM3 sine.100
GPX1 0.00 %
GPX2 0.00 %
GPX3 0.00 %
GPY1 0.00 %
GPY2 0.00 %
GPY3 0.00 %
GPZ1 10.00 %
GPZ2 10.00 %
GPZ3 30.00 %
P16 1000.00 usec

F1 - Acquisition parameters
ND0 1
TD 128
SFO1 500.133 MHz
FIDRES 62.510757 Hz
SW 16.001 ppm
FnMODE QF

F2 - Processing parameters
SI 1024
SF 500.1300119 MHz
WDW SINE
SSB 0
LB 0.00 Hz
GB 0
PC 1.00

F1 - Processing parameters
SI 1024
MC2 QF
SF 500.1311765 MHz
WDW SINE
SSB 0
LB 0.00 Hz
GB 0

2D NMR plot parameters
CX2 18.00 cm
CX1 14.00 cm
F2PLO 8.581 ppm
F2LO 4291.83 Hz
F2PHI 0.023 ppm
F2HI 11.54 Hz
F1PLO 8.335 ppm
F1LO 4168.78 Hz
F1PHI 0.257 ppm
F1HI 128.51 Hz
F2PPMCM 0.47546 ppm/cm
F2HZCM 237.79367 Hz/cm
F1PPMCM 0.57703 ppm/cm
F1HZCM 288.59103 Hz/cm

2-1H

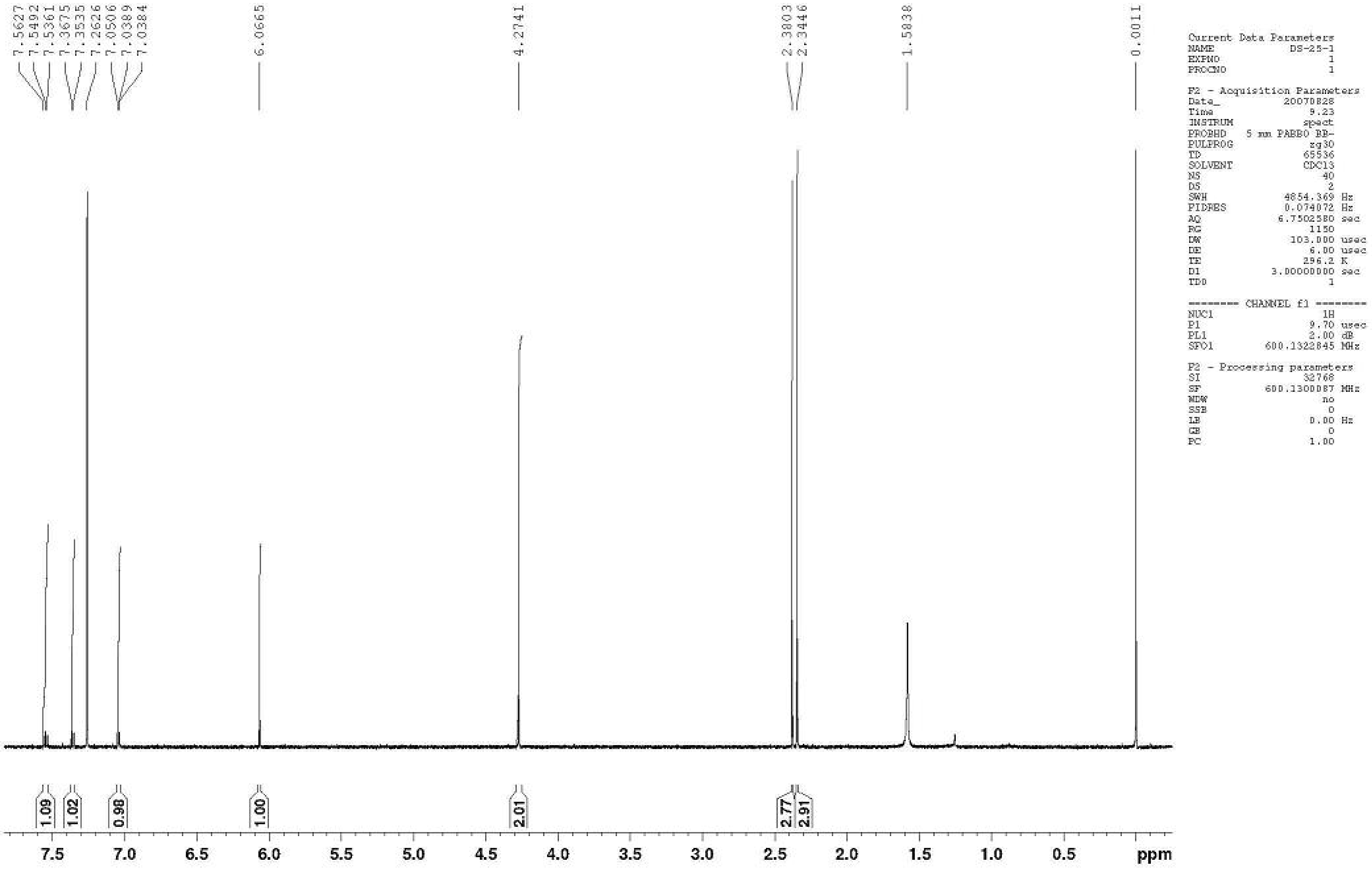

7.5627
7.5492
7.5361
7.3675
7.3535
7.2626
7.0506
7.0389
7.0384
6.0665
4.2741
2.3803
2.3446
1.5838
0.0011
1.09
1.02
0.98
1.00
2.01
2.77
2.91
7.5
7.0
6.5
6.0
5.5
5.0
4.5
4.0
3.5
3.0
2.5
2.0
1.5
1.0
0.5
ppm
Current Data Parameters
NAME DS-25-1
EXPNO 1
PROCNO 1
F2 - Acquisition Parameters
Date_ 20070828
Time 9.23
INSTRUM spect
PROBHD 5 mm PABBO BB-
PULPROG zg30
TD 65536
SOLVENT CDCl3
NS 40
DS 2
SWH 4854.369 Hz
FIDRES 0.074072 Hz
AQ 6.7502580 sec
RG 1150
DW 103.000 usec
DE 6.00 usec
TE 296.2 K
D1 3.00000000 sec
TD0 1
======== CHANNEL f1 ========
NUC1 1H
P1 9.70 usec
PL1 2.00 dB
SFO1 600.1322845 MHz
F2 - Processing parameters
SI 32768
SF 600.1300087 MHz
WDW no
SSB 0
LB 0.00 Hz
GB 0
PC 1.00

2-13C

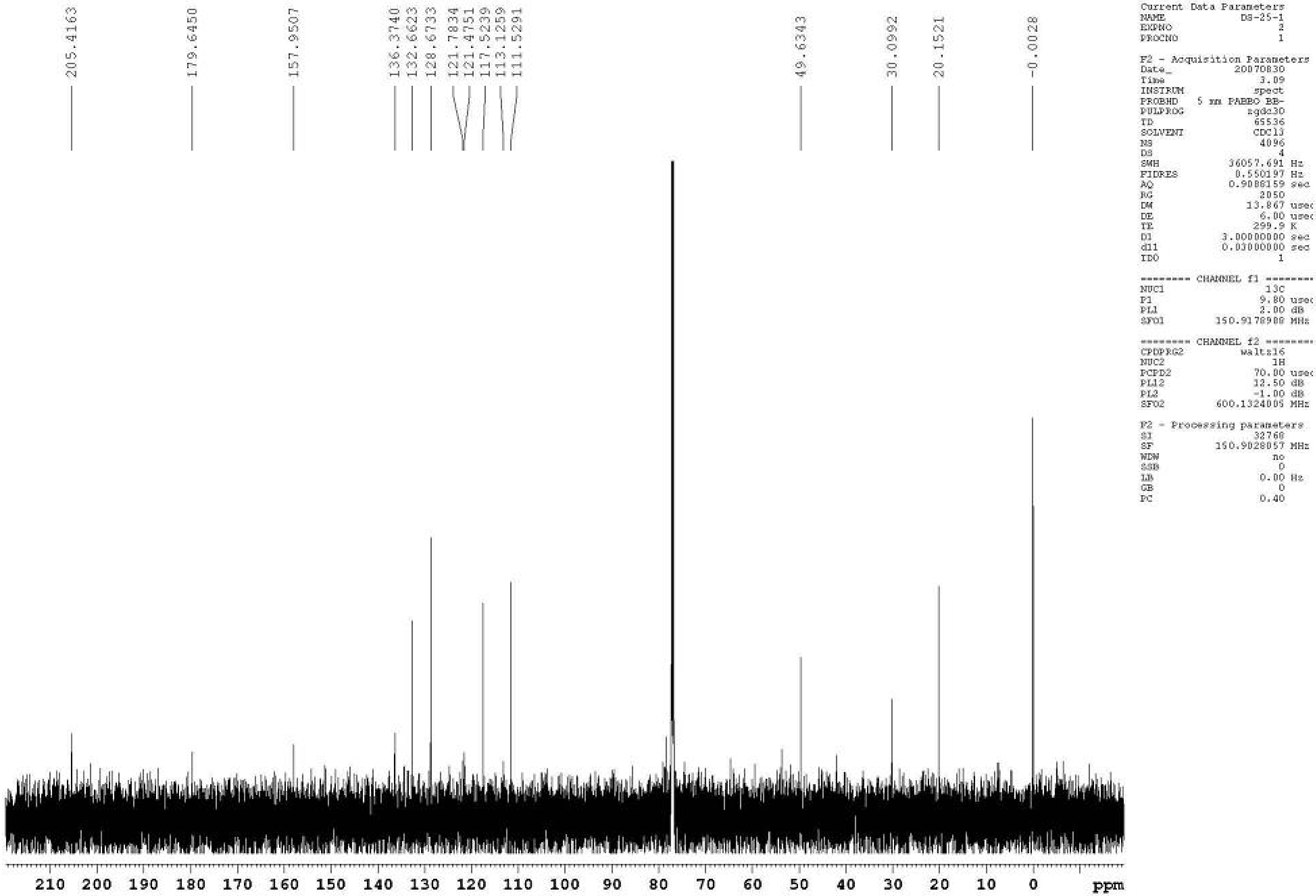
205.4163
179.6450
157.9507
136.3740
132.6623
128.6733
121.7834
121.4751
117.5239
113.1259
111.5291
49.6343
30.0992
20.1521
-0.0028
210 200 190 180 170 160 150 140 130 120 110 100 90 80 70 60 50 40 30 20 10 0 ppm
Current Data Parameters
NAME DS-25-1
EXPNO 2
PROCNO 1
F2 - Acquisition Parameters
Date_ 20070830
Time 3.09
INSTRUM spect
PROBHD 5 mm PABBO BB-
PULPROG zgdc30
TD 65536
SOLVENT CDCl3
NS 4096
DS 4
SWH 36057.691 Hz
FIDRES 0.550197 Hz
AQ 0.9088159 sec
RG 2050
DW 13.867 usec
DE 6.00 usec
TE 299.9 K
D1 3.00000000 sec
d11 0.03000000 sec
TD0 1
======== CHANNEL f1 ========
NUC1 13C
P1 9.80 usec
PL1 2.00 dB
SFO1 150.9178988 MHz
======== CHANNEL f2 ========
CPDPRG2 waltz16
NUC2 1H
PCPD2 70.00 usec
PL12 12.50 dB
PL2 -1.00 dB
SFO2 600.1324005 MHz
F2 - Processing parameters
SI 32768
SF 150.9028057 MHz
WDW no
SSB 0
LB 0.00 Hz
GB 0
PC 0.40

2- H-H- COSY

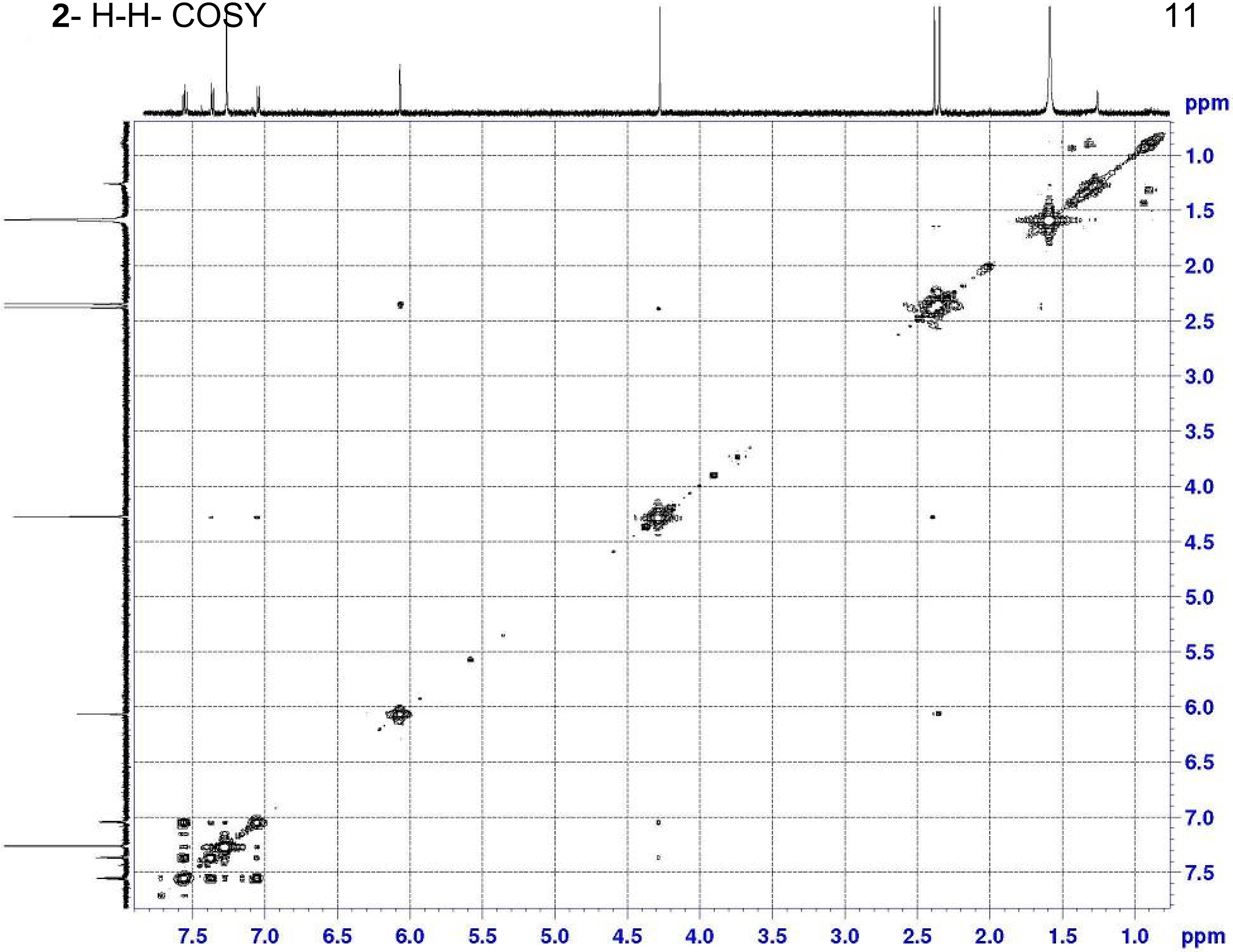
ppm
1.0
1.5
2.0
2.5
3.0
3.5
4.0
4.5
5.0
5.5
6.0
6.5
7.0
7.5
7.5 7.0 6.5 6.0 5.5 5.0 4.5 4.0 3.5 3.0 2.5 2.0 1.5 1.0 ppm

2-HMBC

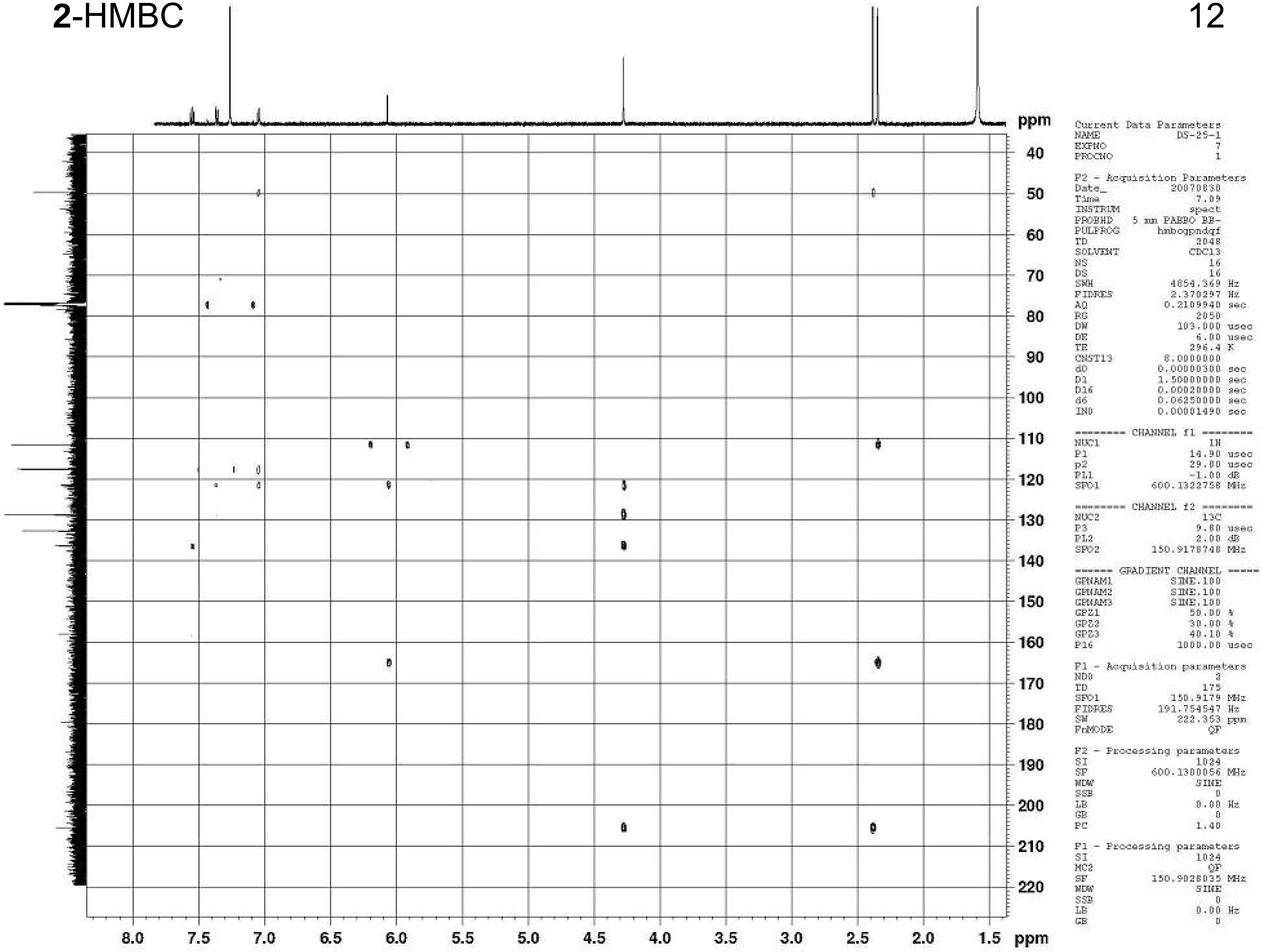
ppm
40
50
60
70
80
90
100
110
120
130
140
150
160
170
180
190
200
210
220
8.0
7.5
7.0
6.5
6.0
5.5
5.0
4.5
4.0
3.5
3.0
2.5
2.0
1.5
ppm
Current Data Parameters
NAME DS-25-1
EXPNO 7
PROCNO 1
F2 - Acquisition Parameters
Date_ 20070830
Time 7.09
INSTRUM spect
PROBHD 5 mm PABBO BB-
PULPROG hmbcgpndqf
TD 2048
SOLVENT CDCl3
NS 16
DS 16
SWH 4854.369 Hz
FIDRES 2.370297 Hz
AQ 0.2109940 sec
RG 2050
DW 103.000 usec
DE 6.00 usec
TE 296.4 K
CNST13 8.0000000
d0 0.00000300 sec
D1 1.50000000 sec
D16 0.00020000 sec
d6 0.06250000 sec
IN0 0.00001490 sec
======== CHANNEL f1 ========
NUC1 1H
P1 14.90 usec
p2 29.80 usec
PL1 -1.00 dB
SFO1 600.1322758 MHz
======== CHANNEL f2 ========
NUC2 13C
P3 9.80 usec
PL2 2.00 dB
SFO2 150.9178748 MHz
====== GRADIENT CHANNEL =====
GPNAM1 SINE.100
GPNAM2 SINE.100
GPNAM3 SINE.100
GPZ1 50.00 %
GPZ2 30.00 %
GPZ3 40.10 %
P16 1000.00 usec
F1 - Acquisition parameters
ND0 2
TD 175
SFO1 150.9179 MHz
FIDRES 191.754547 Hz
SW 222.353 ppm
FnMODE QF
F2 - Processing parameters
SI 1024
SF 600.1300056 MHz
WDW SINE
SSB 0
LB 0.00 Hz
GB 0
PC 1.40
F1 - Processing parameters
SI 1024
MC2 QF
SF 150.9028035 MHz
WDW SINE
SSB 0
LB 0.00 Hz
GB 0

2-HSQC

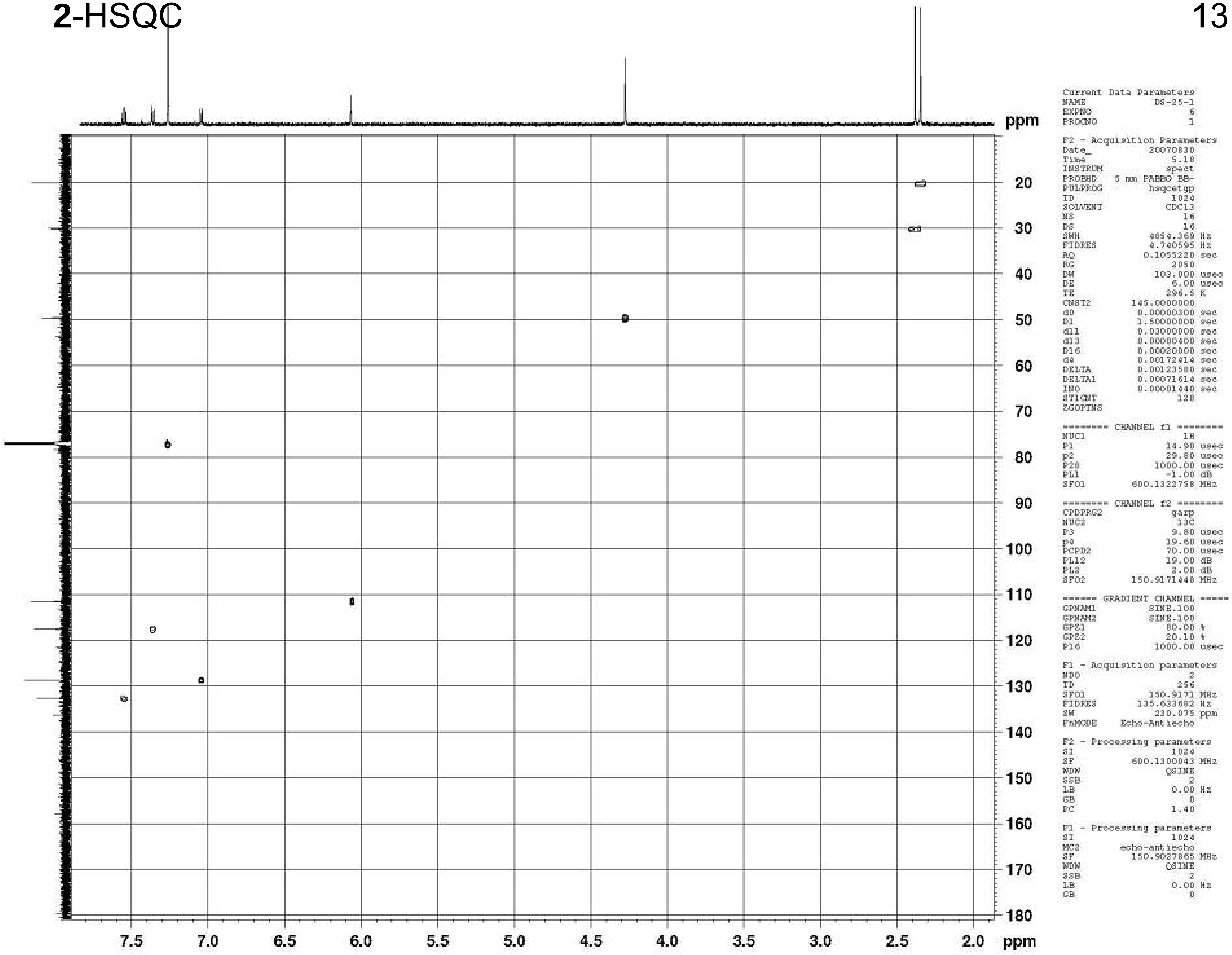
ppm
20
30
40
50
60
70
80
90
100
110
120
130
140
150
160
170
180
7.5
7.0
6.5
6.0
5.5
5.0
4.5
4.0
3.5
3.0
2.5
2.0
ppm
Current Data Parameters
NAME DS-25-1
EXPNO 6
PROCNO 1
F2 - Acquisition Parameters
Date_ 20070830
Time 5.18
INSTRUM spect
PROBHD 5 mm PABBO BB-
PULPROG hsqcetgp
TD 1024
SOLVENT CDCl3
NS 16
DS 16
SWH 4854.369 Hz
FIDRES 4.740595 Hz
AQ 0.1055220 sec
RG 2050
DW 103.000 usec
DE 6.00 usec
TE 296.5 K
CNST2 145.0000000
d0 0.00000300 sec
D1 1.50000000 sec
d11 0.03000000 sec
d13 0.00000400 sec
D16 0.00020000 sec
d4 0.00172414 sec
DELTA 0.00123500 sec
DELTA1 0.00071614 sec
IN0 0.00001440 sec
ST1CNT 128
ZGOPTNS
======== CHANNEL f1 ========
NUC1 1H
P1 14.90 usec
p2 29.80 usec
P28 1000.00 usec
PL1 -1.00 dB
SFO1 600.1322758 MHz
======== CHANNEL f2 ========
CPDPRG2 garp
NUC2 13C
P3 9.80 usec
p4 19.60 usec
PCPD2 70.00 usec
PL12 19.00 dB
PL2 2.00 dB
SFO2 150.9171448 MHz
====== GRADIENT CHANNEL =====
GPNAM1 SINE.100
GPNAM2 SINE.100
GPZ1 80.00 %
GPZ2 20.10 %
P16 1000.00 usec
F1 - Acquisition parameters
ND0 2
TD 256
SFO1 150.9171 MHz
FIDRES 135.633682 Hz
SW 230.075 ppm
FnMODE Echo-Antiecho
F2 - Processing parameters
SI 1024
SF 600.1300043 MHz
WDW QSINE
SSB 2
LB 0.00 Hz
GB 0
PC 1.40
F1 - Processing parameters
SI 1024
MC2 echo-antiecho
SF 150.9027865 MHz
WDW QSINE
SSB 2
LB 0.00 Hz
GB 0

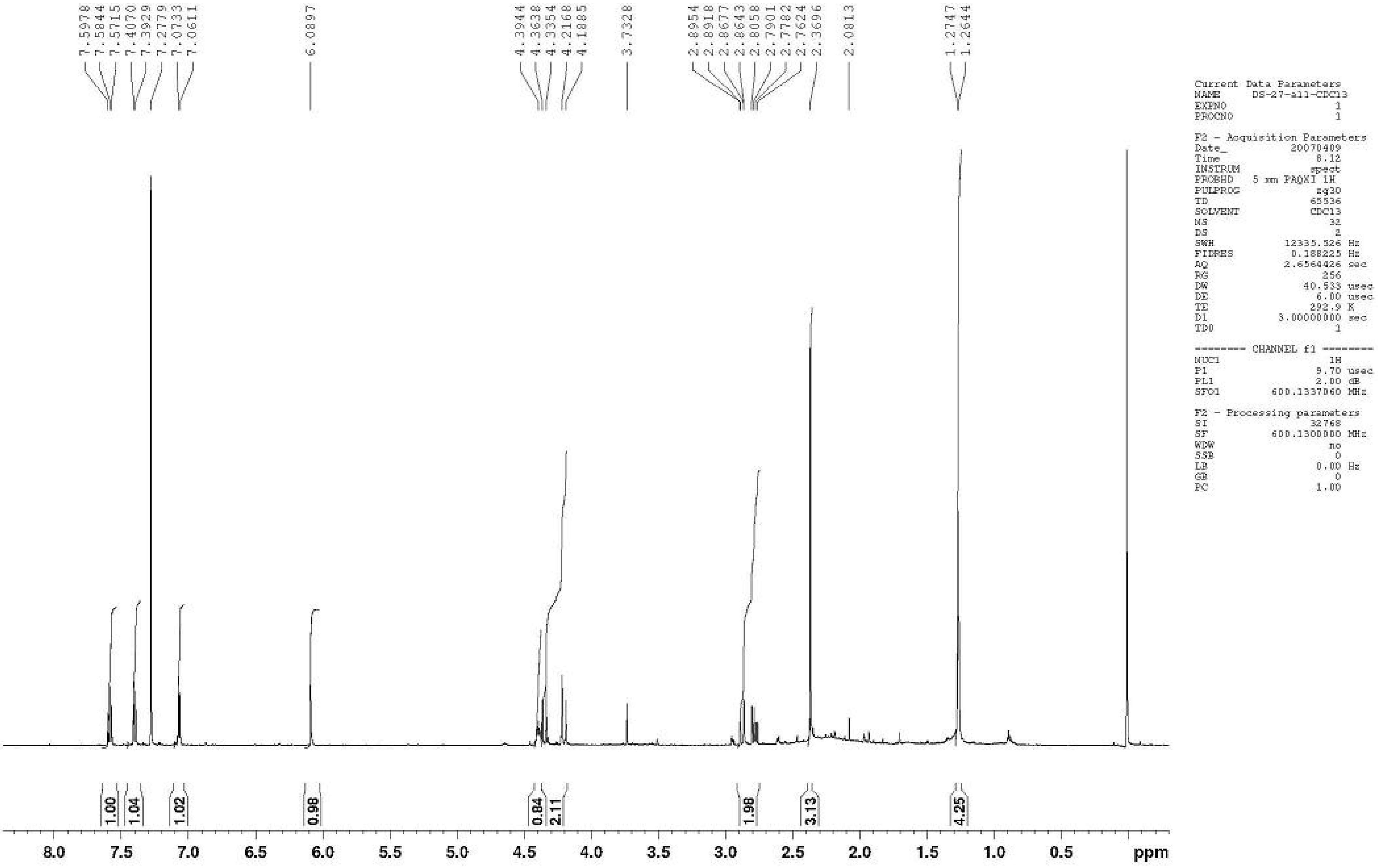
7.5978
7.5844
7.5715
7.4070
7.3929
7.2779
7.0733
7.0611
6.0897
4.3944
4.3638
4.3354
4.2168
4.1885
3.7328
2.8954
2.8918
2.8677
2.8643
2.8058
2.7901
2.7782
2.7624
2.3696
2.0813
1.2747
1.2644
1.00
1.04
1.02
0.98
0.84
2.11
1.98
3.13
4.25
8.0 7.5 7.0 6.5 6.0 5.5 5.0 4.5 4.0 3.5 3.0 2.5 2.0 1.5 1.0 0.5 ppm
Current Data Parameters
NAME DS-27-all-CDCl3
EXPNO 1
PROCNO 1
F2 - Acquisition Parameters
Date_ 20070409
Time 8.12
INSTRUM spect
PROBHD 5 mm PAQXI 1H
PULPROG zg30
TD 65536
SOLVENT CDCl3
NS 32
DS 2
SWH 12335.526 Hz
FIDRES 0.188225 Hz
AQ 2.6564426 sec
RG 256
DW 40.533 usec
DE 6.00 usec
TE 292.9 K
D1 3.00000000 sec
TD0 1
======== CHANNEL f1 ========
NUC1 1H
P1 9.70 usec
PL1 2.00 dB
SFO1 600.1337060 MHz
F2 - Processing parameters
SI 32768
SF 600.1300000 MHz
WDW no
SSB 0
LB 0.00 Hz
GB 0
PC 1.00

3-13C

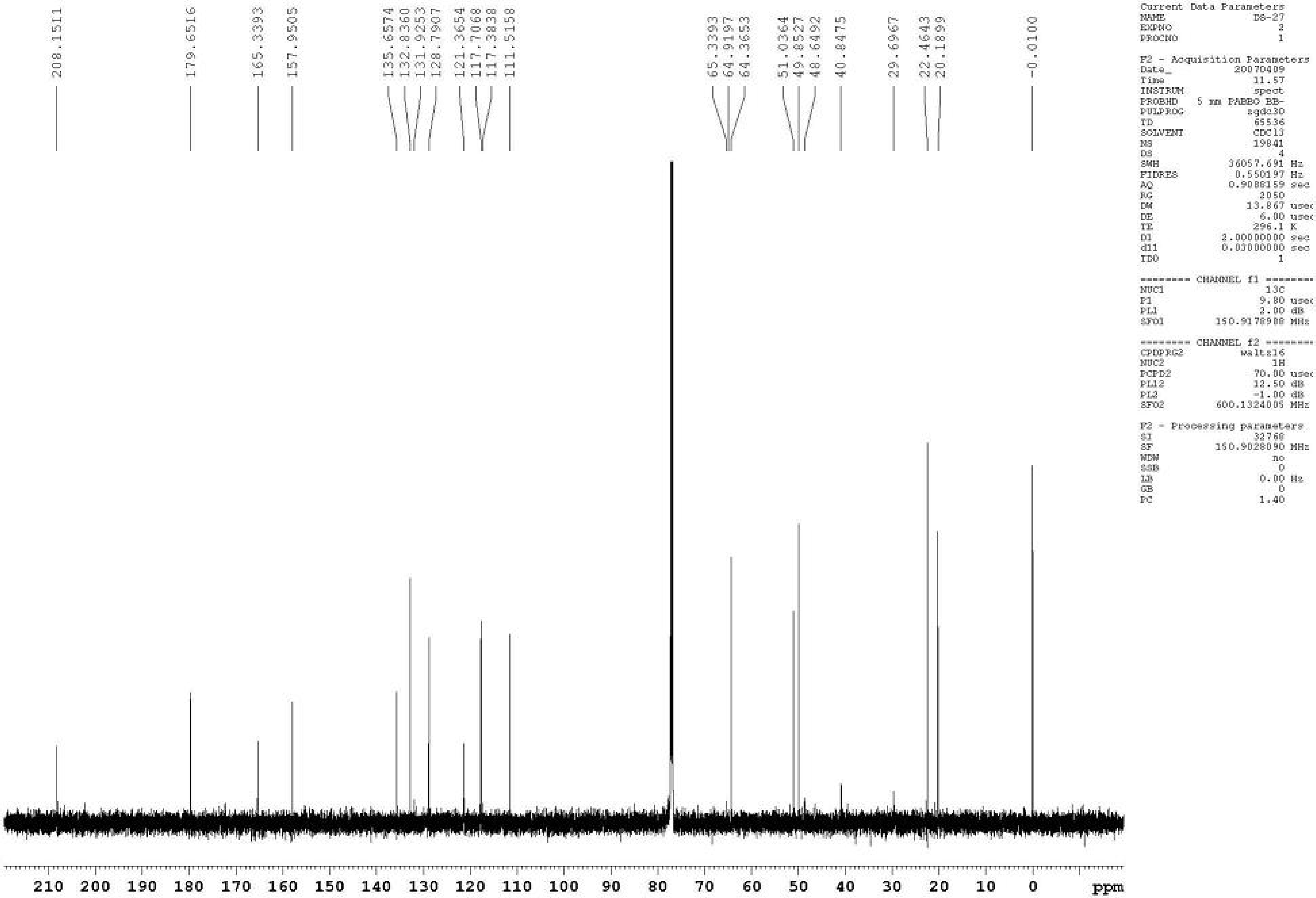
208.1511
179.6516
165.3393
157.9505
135.6574
132.8360
131.9253
128.7907
121.3654
117.7068
117.3838
111.5158
65.3393
64.9197
64.3653
51.0364
49.8527
48.6492
40.8475
29.6967
22.4643
20.1899
-0.0100
210 200 190 180 170 160 150 140 130 120 110 100 90 80 70 60 50 40 30 20 10 0 ppm
Current Data Parameters
NAME DS-27
EXPNO 2
PROCNO 1
F2 - Acquisition Parameters
Date_ 20070409
Time 11.57
INSTRUM spect
PROBHD 5 mm PABBO BB-
PULPROG zgdc30
TD 65536
SOLVENT CDCl3
NS 19841
DS 4
SWH 36057.691 Hz
FIDRES 0.550197 Hz
AQ 0.9088159 sec
RG 2050
DW 13.867 usec
DE 6.00 usec
TE 296.1 K
D1 2.00000000 sec
d11 0.03000000 sec
TD0 1
======== CHANNEL f1 ========
NUC1 13C
P1 9.80 usec
PL1 2.00 dB
SFO1 150.9178988 MHz
======== CHANNEL f2 ========
CPDPRG2 waltz16
NUC2 1H
PCPD2 70.00 usec
PL12 13.50 dB
PL2 -1.00 dB
SFO2 600.1324005 MHz
F2 - Processing parameters
SI 32768
SF 150.9028090 MHz
WDW no
SSB 0
LB 0.00 Hz
GB 0
PC 1.40

3- H-H- COSY

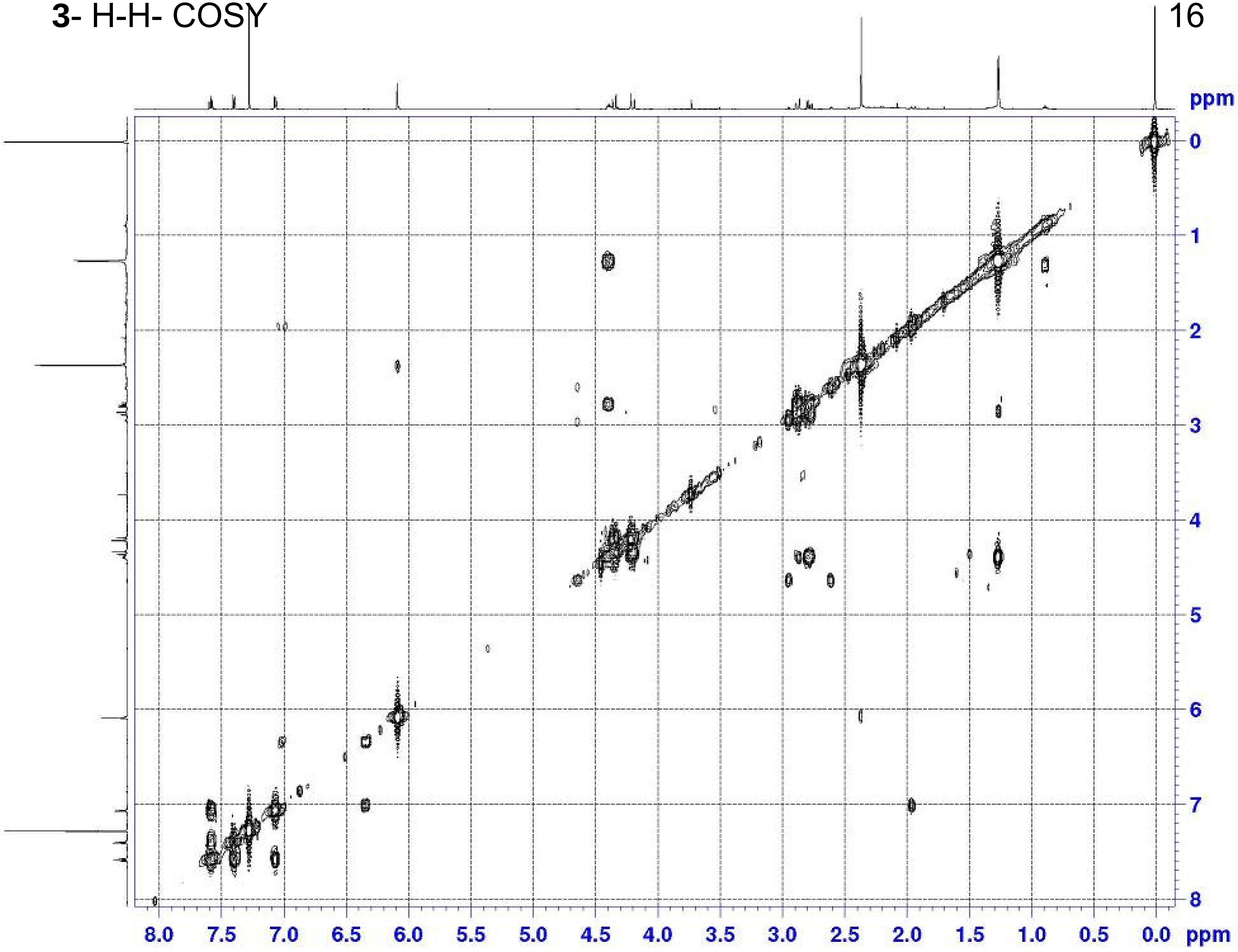

3-HMBC

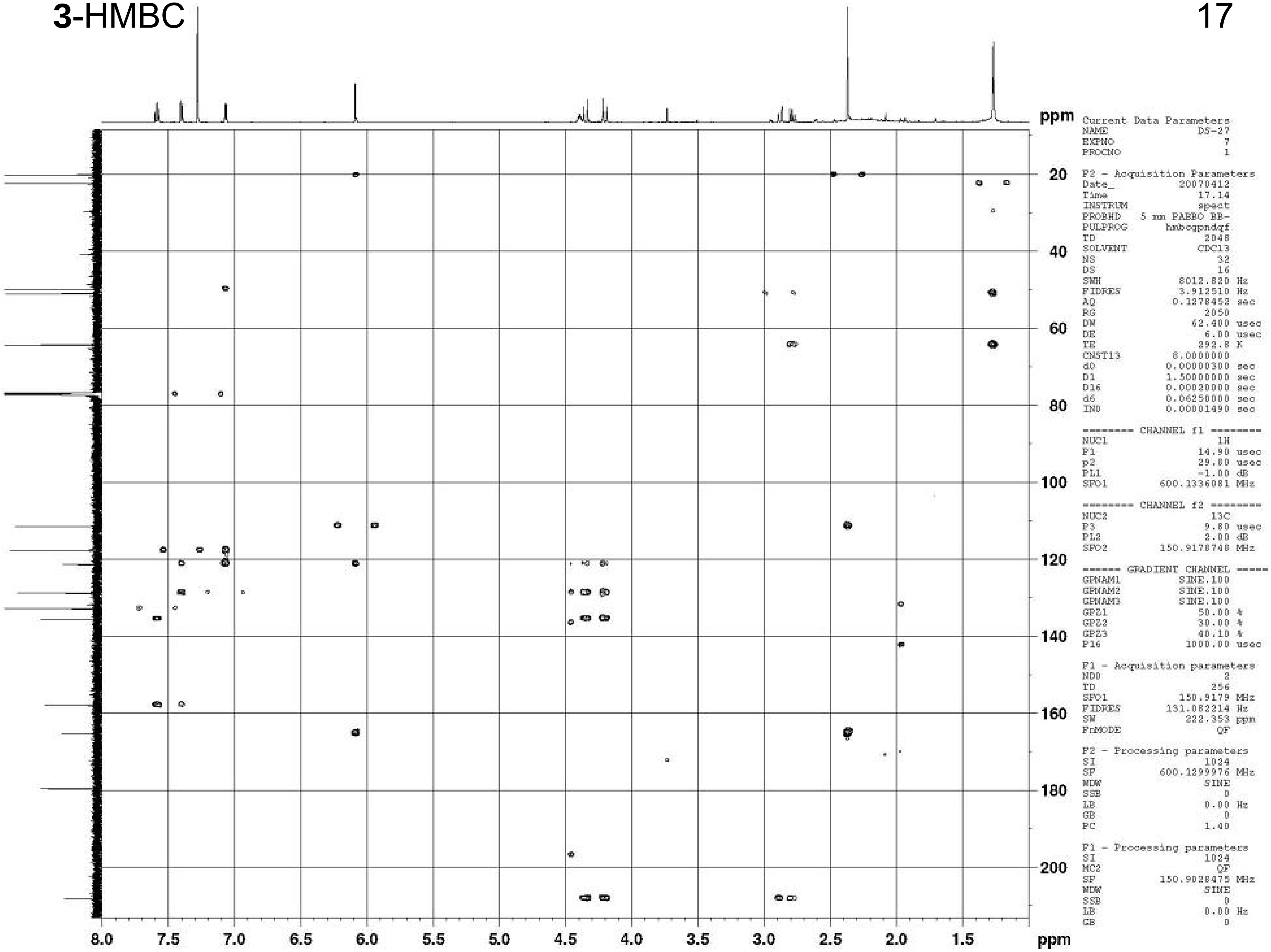

ppm
20
40
60
80
100
120
140
160
180
200
8.0
7.5
7.0
6.5
6.0
5.5
5.0
4.5
4.0
3.5
3.0
2.5
2.0
1.5
ppm
Current Data Parameters
NAME DS-27
EXPNO 7
PROCNO 1
F2 - Acquisition Parameters
Date_ 20070412
Time 17.14
INSTRUM spect
PROBHD 5 mm PABBO BB-
PULPROG hmbcgpndqf
TD 2048
SOLVENT CDCl3
NS 32
DS 16
SWH 8012.820 Hz
FIDRES 3.912510 Hz
AQ 0.1278452 sec
RG 2050
DW 62.400 usec
DE 6.00 usec
TE 292.8 K
CNST13 8.0000000
d0 0.00000300 sec
D1 1.50000000 sec
D16 0.00020000 sec
d6 0.06250000 sec
IN0 0.00001490 sec
======== CHANNEL f1 ========
NUC1 1H
P1 14.90 usec
p2 29.80 usec
PL1 -1.00 dB
SFO1 600.1336081 MHz
======== CHANNEL f2 ========
NUC2 13C
P3 9.80 usec
PL2 2.00 dB
SFO2 150.9178748 MHz
====== GRADIENT CHANNEL =====
GPNAM1 SINE.100
GPNAM2 SINE.100
GPNAM3 SINE.100
GPZ1 50.00 %
GPZ2 30.00 %
GPZ3 40.10 %
P16 1000.00 usec
F1 - Acquisition parameters
ND0 2
TD 256
SFO1 150.9179 MHz
FIDRES 131.082214 Hz
SW 222.353 ppm
FnMODE QF
F2 - Processing parameters
SI 1024
SF 600.1299976 MHz
WDW SINE
SSB 0
LB 0.00 Hz
GB 0
PC 1.40
F1 - Processing parameters
SI 1024
MC2 QF
SF 150.9028475 MHz
WDW SINE
SSB 0
LB 0.00 Hz
GB 0

3-HSQC

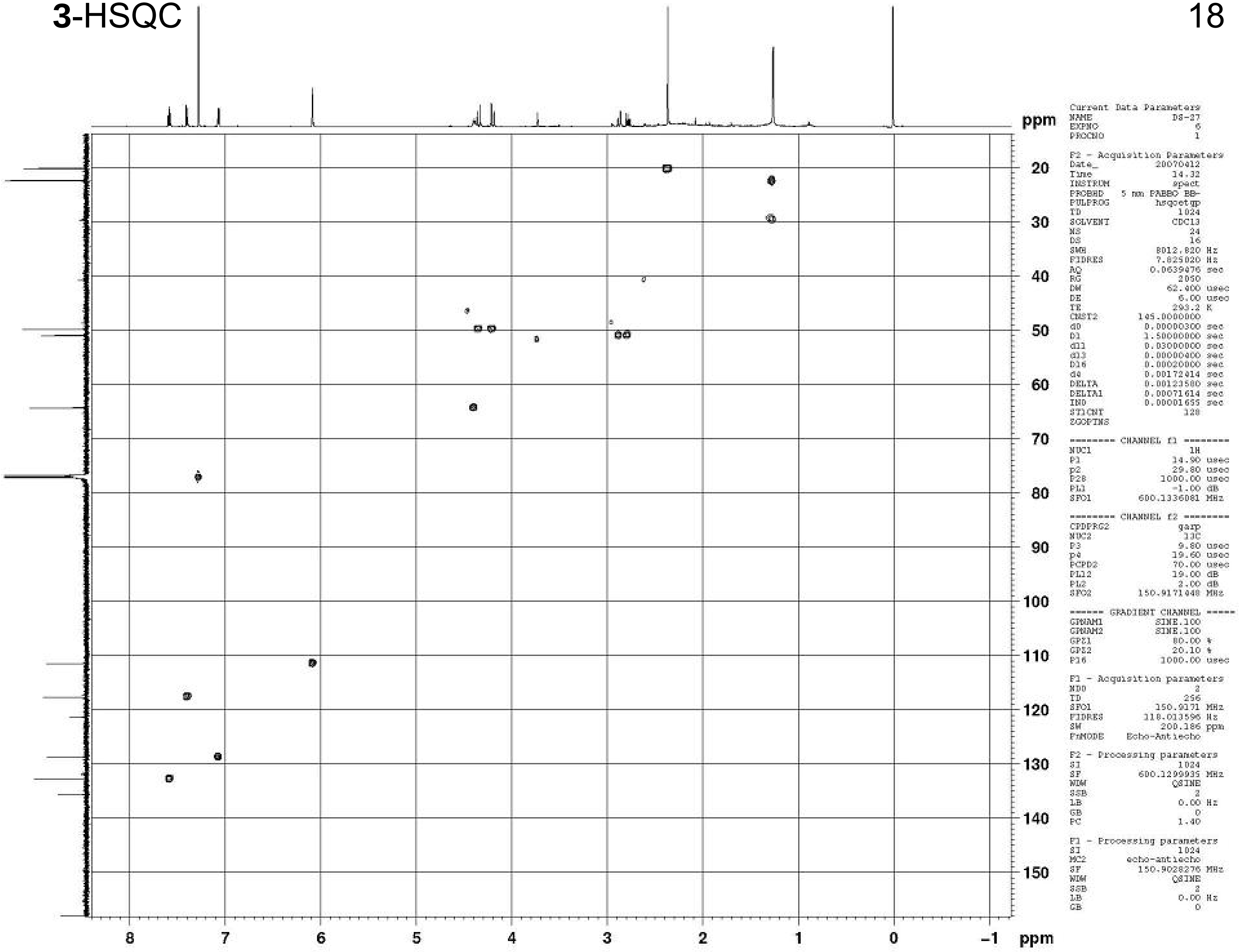
ppm
20
30
40
50
60
70
80
90
100
110
120
130
140
150
8
7
6
5
4
3
2
1
0
–1
ppm
Current Data Parameters
NAME DS-27
EXPNO 6
PROCNO 1
F2 - Acquisition Parameters
Date_ 20070412
Time 14.32
INSTRUM spect
PROBHD 5 mm PABBO BB-
PULPROG hsqcetgp
TD 1024
SOLVENT CDCl3
NS 24
DS 16
SWH 8012.820 Hz
FIDRES 7.825020 Hz
AQ 0.0639476 sec
RG 2050
DW 62.400 usec
DE 6.00 usec
TE 293.2 K
CNST2 145.0000000
d0 0.00000300 sec
D1 1.50000000 sec
d11 0.03000000 sec
d13 0.00000400 sec
D16 0.00020000 sec
d4 0.00172414 sec
DELTA 0.00123580 sec
DELTA1 0.00071614 sec
IN0 0.00001655 sec
ST1CNT 128
ZGOPTNS
======== CHANNEL f1 ========
NUC1 1H
P1 14.90 usec
p2 29.80 usec
P28 1000.00 usec
PL1 -1.00 dB
SFO1 600.1336081 MHz
======== CHANNEL f2 ========
CPDPRG2 garp
NUC2 13C
P3 9.80 usec
p4 19.60 usec
PCPD2 70.00 usec
PL12 19.00 dB
PL2 2.00 dB
SFO2 150.9171948 MHz
====== GRADIENT CHANNEL =====
GPNAM1 SINE.100
GPNAM2 SINE.100
GPZ1 80.00 %
GPZ2 20.10 %
P16 1000.00 usec
F1 - Acquisition parameters
ND0 2
TD 256
SFO1 150.9171 MHz
FIDRES 118.013596 Hz
SW 200.186 ppm
FnMODE Echo-Antiecho
F2 - Processing parameters
SI 1024
SF 600.1299935 MHz
WDW QSINE
SSB 2
LB 0.00 Hz
GB 0
PC 1.40
F1 - Processing parameters
SI 1024
MC2 echo-antiecho
SF 150.9028276 MHz
WDW QSINE
SSB 2
LB 0.00 Hz
GB 0

HPLC data of compound **1** and crude extract

Compound **1** was examined by reversed-phase HPLC on a 4.6 × 250 mm C-18 (5 μm) column (Merck) with CH_3CN from 50% to 100% in 15 min (1 mL/min flow rate, 254 nm detection) with a retention time of 11.3 min. Crude extract was examined by the same method. The peak of compound **1** was detected with a retention time of 11.3 min.

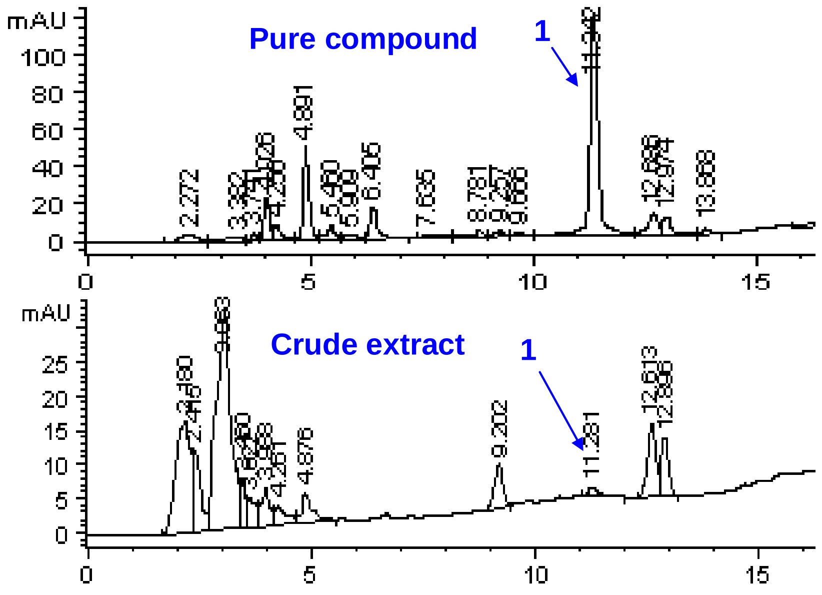

Figure 1: The HPLC result of compound **1** and crude extract.
